# Zanubrutinib-based regimen as the salvage or bridging treatment of CART therapy in relapsed or refractory, non-germinal center B-cell–like diffuse large B-cell lymphoma: a retrospective multicenter cohort study

**DOI:** 10.3389/fimmu.2026.1806191

**Published:** 2026-04-07

**Authors:** Yan Lu, Shiguang Ye, Lili Zhou, Xianggui Yuan, Ping Li, Wenbin Qian, Aibin Liang

**Affiliations:** 1 Department of Hematology, Tongji Hospital of Tongji University, Shanghai, China; 2 Department of Hematology, The Second Affiliated Hospital, College of Medicine, Zhejiang University, Hangzhou, Zhejiang, China

**Keywords:** BTK inhibitor, CAR-T therapy, non-GCB DLBCL, relapsed or refractory DLBCL, zanubrutinib-combined therapy

## Abstract

**Background:**

Relapsed or refractory (R/R) non–germinal center B-cell–like (non-GCB) diffuse large B-cell lymphoma (DLBCL) shows poor clinical outcomes. Bruton tyrosine kinase (BTK) inhibitors have established therapeutic activity by targeting B-cell receptor signaling, with promising results in treating DLBCL. The monotherapy of zanubrutinib, a selective BTK inhibitor, or second-line salvage chemotherapy has shown limited efficacy in patients with R/R DLBCL. Thus, the present study evaluated the efficacy and safety of zanubrutinib-combined therapy in heavily treated patients with non-GCB DLBCL.

**Methods:**

This retrospective study consists of 27 heavily treated patients with non-GCB DLBCL who received zanubrutinib-combined therapy between January 2021 and February 2024 in Shanghai Tongji Hospital and Zhejiang Second Affiliated Hospital. Efficacy outcomes included overall response rate (ORR), progression-free survival (PFS), and overall survival (OS), whereas safety outcomes included incidence of adverse events.

**Results:**

Of all the 27 enrolled patients’ baseline,24 patients (88.9%) showed a high IPI score (≥3), 23 patients (85.2%) had a high proliferation score (Ki 67≥80%) and 20 patients (74.1%) were heavily treated with ≥3 lines of previous treatments. The ORR in all patients was 74.1% (95%CI, 53.7%-88.9%), the partial response (PR) was 66.7% (95%CI, 46.0%-83.5%). With a median follow-up of 36.6 months, the median PFS was 10.6 months (95%CI 7.3-14) and median OS was 19.6 months (95%CI 12.3-not reached). The grade ≥3 hematologic toxicities included neutropenia (85.1%, 23/27) and thrombocytopenia (37%, 10/27). The grade ≥3 nonhematologic AEs were hypokalemia (11.1%, 3/27) and pulmonary infection (11.1%, 3/27). No treatment-related deaths occurred. Subgroups stratified by gender, age, and presence/absence of extranodal lesions all maintained an ORR of over 70%. The efficacy of the combined therapy seemed to be not affected by most baseline characteristics and was associated with high response even in high-risk subgroups. Of all the evaluated 27 patients, 2 patients got complete response (CR) received autologous stem cell transplantation and lenalidomide maintenance therapy respectively. 19 patients got no CR were bridged to CD19 chimeric antigen receptor (CAR)-T cell therapy, while the other 6 patients received additional salvage chemotherapy. In the CAR-T cohort, the ORR was 89.5% (95%CI: 67.0%~98.2%) and CR was 57.9% (95%CI: 34.5%~78.9%), the median PFS was 14 months (95% CI: 5.2-37.9) and median OS was 27.7 months (95% CI: 10.1- not reached). The CAR-T group was associated with improved overall survival relative to the non-CAR-T group (HR = 0.21, 95% CI: 0.05–0.79, P = 0.02). In the landmark analysis, the survival probability of CART group was 80% at 12 months post-landmark, the non-CAR-T group exhibited an earlier initial drop with survival decreasing to 57.1% at 12 months.

**Conclusion:**

Zanubrutinib-combined therapy was effective and safe for the treatment of heavily treated patients with non-GCB DLBCL. It offers a promising treatment option and serving as an effective bridge to CAR-T therapy, with manageable toxicity. Future prospective studies with larger cohorts are needed to validate these findings.

## 1 Introduction

Non-Hodgkin lymphoma (NHL) is the seventh most common cancer globally, with diffuse large B-cell lymphoma (DLBCL) accounting for 30%-40% of adult NHL cases in Western countries and 40% in China (1–3). Based on cell-of-origin and immunohistochemical profiles (Hans’ algorithm), DLBCL is classified into germinal center B-cell–like (GCB) and non-GCB subtypes, with the latter showing higher aggressiveness, chemoresistance, and recurrence rates (4, 5). Rituximab plus CHOP (R-CHOP) has been the first-line standard for over a decade, but 4%-12% of patients are primary refractory, and 17%-40% relapse after initial response (6–8). Salvage chemotherapy and autologous stem cell transplantation (ASCT) yield limited outcomes that the overall response rate (ORR) is 55%-70% (3-year event-free survival: 21%), and CAR-T therapy is restricted by accessibility and toxicity (9, 10). BTK, a key component of B-cell receptor signaling, is a validated target for non-GCB DLBCL (11, 12). The first generation BTK inhibitors (ibrutinib, acalabrutinib) show modest ORR (23%-24%), while zanubrutinib—a highly selective next-generation BTK inhibitor—exhibits reduced off-target activity and better tolerability (13–15). However, zanubrutinib monotherapy (ORR: 29.3%) and salvage therapy alone have limited efficacy, prompting evaluation of combined regimens (14). This study investigates the efficacy and safety of zanubrutinib-combined therapy in heavily treated R/R non-GCB DLBCL patients in China.

## 2 Materials and methods

### 2.1 Study design and patients

This retrospective multicenter study included 27 R/R non-GCB DLBCL patients treated with zanubrutinib-based regimens in Shanghai Tongji Hospital and Zhejiang Second Affiliated Hospital between January 2021 and February 2024. Inclusion criteria: age >18 years; histopathologically confirmed non-GCB DLBCL (Hans’ algorithm); assessable lesions (CT/MRI); and zanubrutinib-containing treatment. Exclusion criteria: severe complications/comorbidities, active infection, uncontrolled bleeding, or pregnancy/breastfeeding. The study was approved by institutional review boards and conducted in compliance with Good Clinical Practice, ICH guidelines, and the Declaration of Helsinki. All patients provided written informed consent in this retrospective study participation.

### 2.2 Treatment

All patients received zanubrutinib (Zan, 160 mg twice daily) until disease progression or intolerance. Three treatment regimens were used: (1) Zan + LEN (lenalidomide) ± rituximab; (2) Zan + chemotherapy (EPOCH, ICE, Gemox, ESHAP, CHOPE, GDP) ± rituximab; (3) Zan + chemotherapy + LEN ± rituximab. Disease evaluation was performed after at least 2 cycles of the Zan-based treatment. The patient got complete response (CR) and eligible to transplant would be recommended to receive auto-HSCT. The patient got CR and ineligible or defer transplant would be recommended to receive LEN maintenance. The patient got partial response (PR) would be recommended to continue the Zan-based regimen or CD19-CAR-T therapy. The patient got stable disease (SD) or progressive disease (PD) would be recommended to receive CD19-CAR-T therapy or other salvage chemotherapy.

### 2.3 Treatment outcomes

Efficacy was assessed by CT scan every 2 cycles (6 weeks) after treatment initiation, and PET-CT was performed after 3 cycles or confirmed complete response. Efficacy was assessed per Lugano 2014 Criteria (16): ORR (CR+PR), CR, PR, progression-free survival (PFS), overall survival (OS), and duration of response (DOR). Adverse events (AEs) were graded per CTCAE v5.0. The overall OS was defined as the time from Zan-based therapy treatment to death or last follow-up. Patients who were lost to follow-up or were alive at the date of data cutoff were censored at the last date they were known to be alive. The overall PFS was defined as the time from Zan-based therapy treatment to the first occurrence of disease progression as determined by the investigator (according to Lugano 2014 Criteria) or death from any cause, whichever occurred first. The OS and PFS for subsequent CART group or non-CART group was also defined from the initial time from Zan-based therapy treatment. The day of the last follow-up was January, 31th, 2025.

### 2.4 Statistical analysis

Statistical analyses were performed using Prism 9.0 (GraphPad, CA, USA) and R software (version 4.2.1; R Foundation for Statistical Computing, Vienna, Austria). Survival outcomes, including DOR, PFS and OS, were estimated using the Kaplan-Meier method and compared using the log-rank test, implemented with the survival package. P < 0.05 was significant. To evaluate the impact of response status on subsequent survival while avoiding guarantee-time bias, a landmark analysis was conducted. Patients who were alive and event−free at the landmark time were stratified according to their response status at that time (responders vs. non−responders), and survival from the landmark time onward was compared between groups using Cox proportional hazards models, also fitted with the survival package. To identify independent prognostic factors associated with survival, multivariable Cox proportional hazards regression models were fitted using the coxph() function in the survival package. Variables with a P value < 0.10 in the univariable analysis, along with clinically relevant factors, were entered into the multivariable model. The final model was selected using a backward stepwise elimination procedure based on the Akaike Information Criterion (AIC). The proportional hazards assumption was tested by examining Schoenfeld residuals. All tests were two−sided, and a P value < 0.05 was considered statistically significant. Categorical variables were reported as percentages (Fisher’s exact test). Efficacy outcomes were presented with 95% confidence intervals (CI).

## 3 Results

### 3.1 Baseline demographic and clinical characteristics

Of 27 enrolled patients, median age was 58 years (range: 38-76); 19 (70.4%) were male. Median prior treatment lines: 3 (range: 2-7). Baseline features included: International Prognostic Index (IPI) score ≥3 (88.9%), extranodal involvement (92.6%), bulky disease (≥10 cm, 33.3%), TP53 mutation (18.5%), and C-myc rearrangement (22.2%) (**Table 1**). Patients were received one of the three zanubrutinib-combined regimens: (1) Zan + LEN ± rituximab (5 patients), (2) Zan + chemotherapy ± rituximab (17 patients), and (3) Zan + chemotherapy + LEN ± rituximab (5 patients).

**Table 1:** Baseline Characteristics of All 27 Treated Patients.

Characteristic	Patients
Median age (range) — years	58 (38-76)
≥ 65years — no. (%)	13 (48.2)
< 65years — no. (%)	14 (51.8)
Sex	
Male — no. (%)	19 (70.4)
Female — no. (%)	8 (29.6)
Patients complicated with underling diseases	
Diabetes — no. (%)	6 (22.2)
Cardiopathy — no. (%)	3 (11.1)
COPD — no. (%)	3 (11.1)
Primary refractory disease — no. (%)	23 (85.2)
Relapsed disease — no. (%)	4 (14.8)
ECOG	
≥2 — no. (%)	17 (63)
<2 — no. (%)	10 (37)
Intermediate or high risk according to simplified IPI	
≥3 — no. (%)	24 (88.9)
<3 — no. (%)	3 (11.1)
Ki-67 proliferation index	
≥80% — no. (%)	23 (85.2)
<80% — no. (%)	4 (14.8)
TP53 deletion / mutations	
Yes — no. (%)	5 (18.5)
No — no. (%)	12 (44.4)
Unknown — no. (%)	10 (37.1)
C-MYC rearrangement	
Yes — no. (%)	6 (22.2)
No — no. (%)	14 (51.9)
Unknown — no. (%)	7 (25.9)
Number of previous therapies, Median (range)	3 (2-7)
≥3 Previous lines of therapy — no. (%)	20 (74.1)
<3 Previous lines of therapy — no. (%)	7 (25.9)
Extranodal disease	
Yes — no. (%)	25 (92.6)
No — no. (%)	2 (7.4)
Bulky disease	
diameter ≥ 5cm — no. (%)	18 (66.7)
diameter ≥ 10cm — no. (%)	9 (33.3)

### 3.2 Treatment and outcome

**Figure 1:**
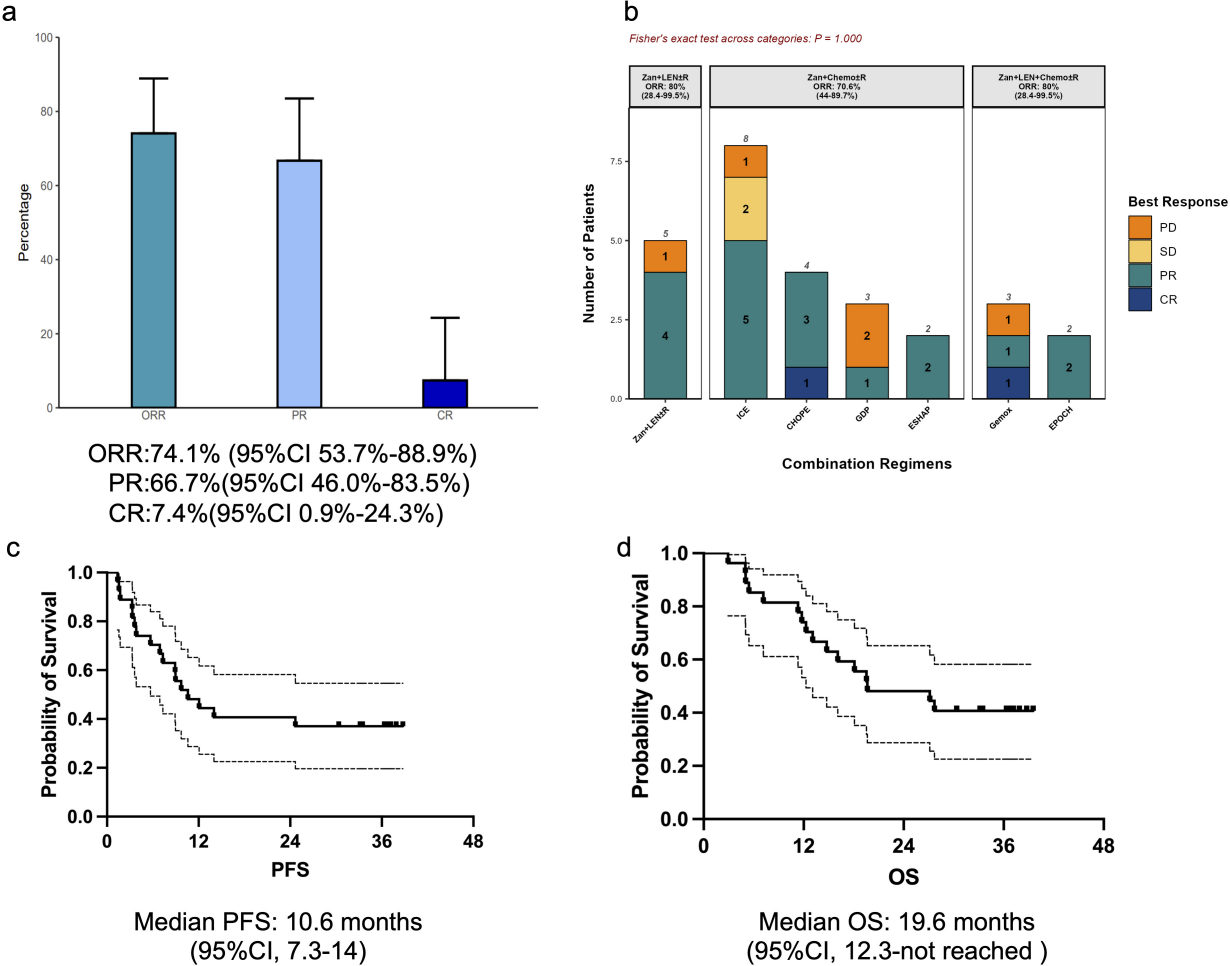
Overall response of zanubrutinib-combined therapy. **(a)** Response rates of all evaluable patients (CR, complete response; PR, partial response; SD, stable disease; PD, progressive disease); **(b)** The distribution and ORR of the three group regimens; **(c)** Kaplan-Meier curve for progression-free survival (PFS); **(d)** Kaplan-Meier curve for overall survival (OS).

Overall ORR was 74.1% (95%CI: 53.7%-88.9%), with CR (7.4%, 95%CI: 0.9%-24.3%) and PR (66.7%, 95%CI: 46.0%-83.5%) (**Figure 1a**). The ORR of the three regimens (Zan + LEN ± Rituximab, Zan + chemotherapy ± rituximab and Zan + chemotherapy + LEN ± rituximab) was respectively 80% (4/5, 95%CI 28.4%-99.5%), 70.6% (12/17; 95% CI 44.0% - 89.7%) and 80% (4/5, 95%CI 28.4%-99.5%) (**Figure 1b**). The Fisher’s exact test across the 3 groups show no significant difference in the efficacy (P value is 1). Median follow-up was 36.6 months; median PFS was 10.6 months (95%CI, 7.3-14) and median OS was 19.6 months (95%CI, 12.3-not reached) (**Figures 1c, d**). Median treatment duration of Zan-based regimen was 5.2 months (range: 1.5-14).

### 3.3 Adverse events

All 27 evaluable patients reported treatment-related AEs. Grade ≥3 hematologic AEs: neutropenia (85.1%, 23/27) and thrombocytopenia (37%, 10/27). Grade ≥3 nonhematologic AEs: hypokalemia (11.1%, 3/27) and pulmonary infection (11.1%, 3/27). Management for grade ≥3 hypokalemia followed institutional guidelines: received intravenous potassium chloride supplementation under continuous cardiac monitoring. The causes of grade ≥3 hypokalemia was associated with diuretics and chemotherapy-induced asitia. Management for grade ≥3 pulmonary infection is empiric broad-spectrum antibiotics and antifungal therapy in patients with persistent fever and neutropenia. Concomitant anti-cancer therapy (e.g., zanubrutinib) was temporarily withheld until the infection resolved to ≤ grade 1. Granulocyte colony-stimulating factor (G-CSF) was administered to patients with febrile neutropenia. No treatment-related deaths occurred (**Table 2**).

**Table 2:** Treatment-related adverse events.

Event number of patients (percent)	Any grade	Grade 1	Grade 2	Grade 3	Grade 4
Neutropenia	25(92.5)	1(3.7)	1(3.7)	11(40.7)	12(44.4)
Thrombocytopenia	23(85.2)	8(29.6)	5(18.6)	3(11.1)	7(25.9)
Anemia	17(63.0)	8(29.6)	1(3.7)	8(29.6)	0(0)
Hypoalbuminemia	11(40.7)	5(18.5)	6(22.2)	0(0)	0(0)
Hypokalemia	15(55.5)	6(22.2)	6(22.2)	3(11.1)	0(0)
AST/ALT increased	9(33.3)	7(25.9)	2(7.4)	0(0)	0(0)
TBil increased	7(26.0)	5(18.6)	2(7.4)	0(0)	0(0)
Arrhythmia	4(14.8)	4(14.8)	0(0)	0(0)	0(0)
Infection	12(44.4)	4(14.8)	5(18.5)	3(11.1)	0(0)

### 3.4 Subgroup analysis

**Figure 2:**
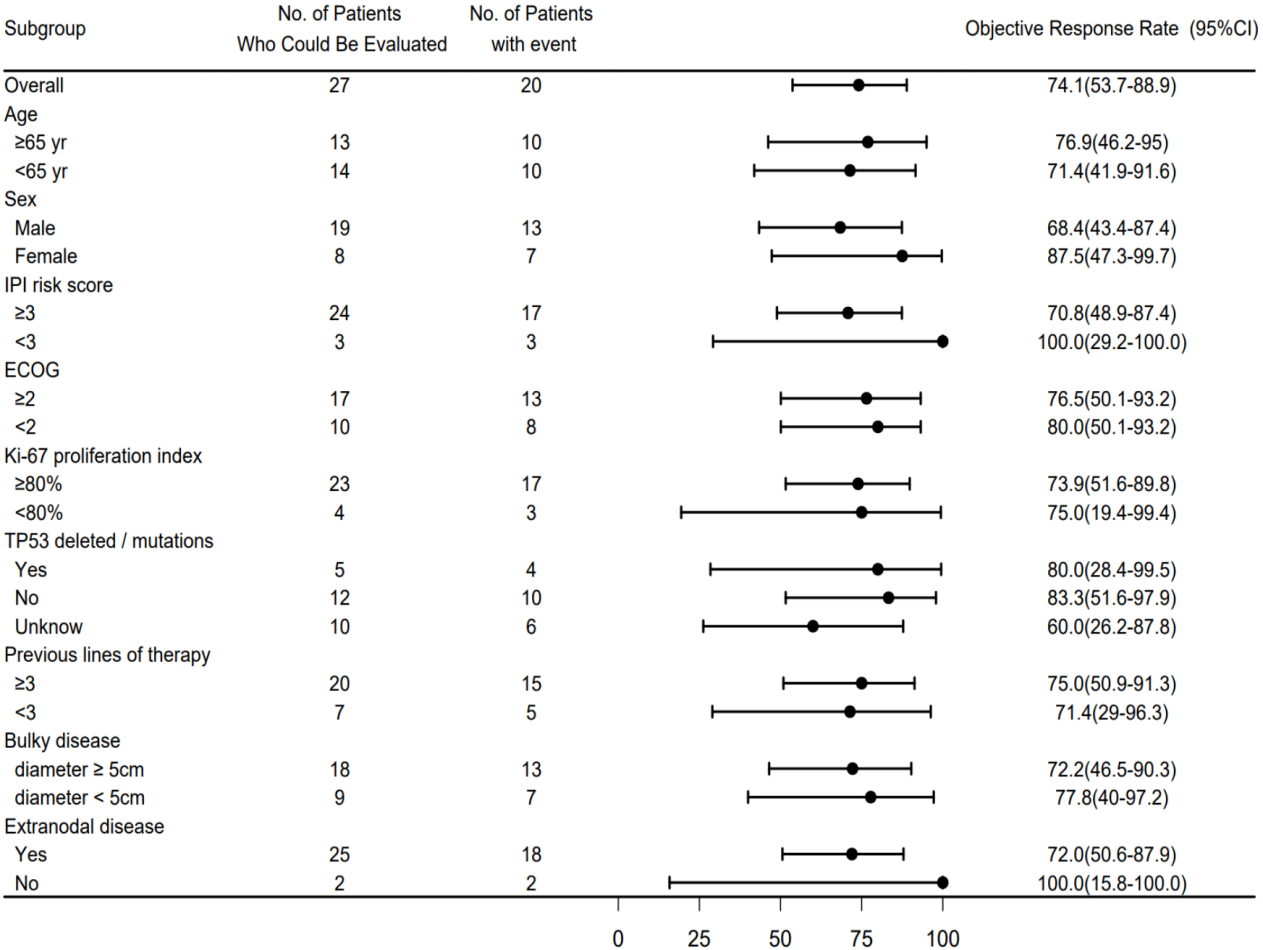
Subgroup analysis of overall response rate (ORR).

Subgroups stratified by gender, age (≥65 years vs. <65 years), and presence/absence of extranodal lesions all maintained an ORR of over 70% (**Figure 2**). The efficacy of the combined therapy seemed to be not affected by most baseline characteristics and was associated with high response even in high-risk subgroups. In high-risk feature subgroups: For patients with an IPI score ≥3 (88.9% of all patients), the ORR reached 70.8%; for those with “Ki-67” ≥80% (85.2% of all patients), the ORR was 73.9%; and for patients who had received ≥3 lines of previous treatment (74.1% of all patients), the ORR reached 75%. In genetic and clinical feature subgroups: The ORR was 80% in patients with TP53 mutations and 83.3% in those without mutations.

### 3.5 Outcome of subsequent CD19 CAR-T or other therapy

**Figure 3:**
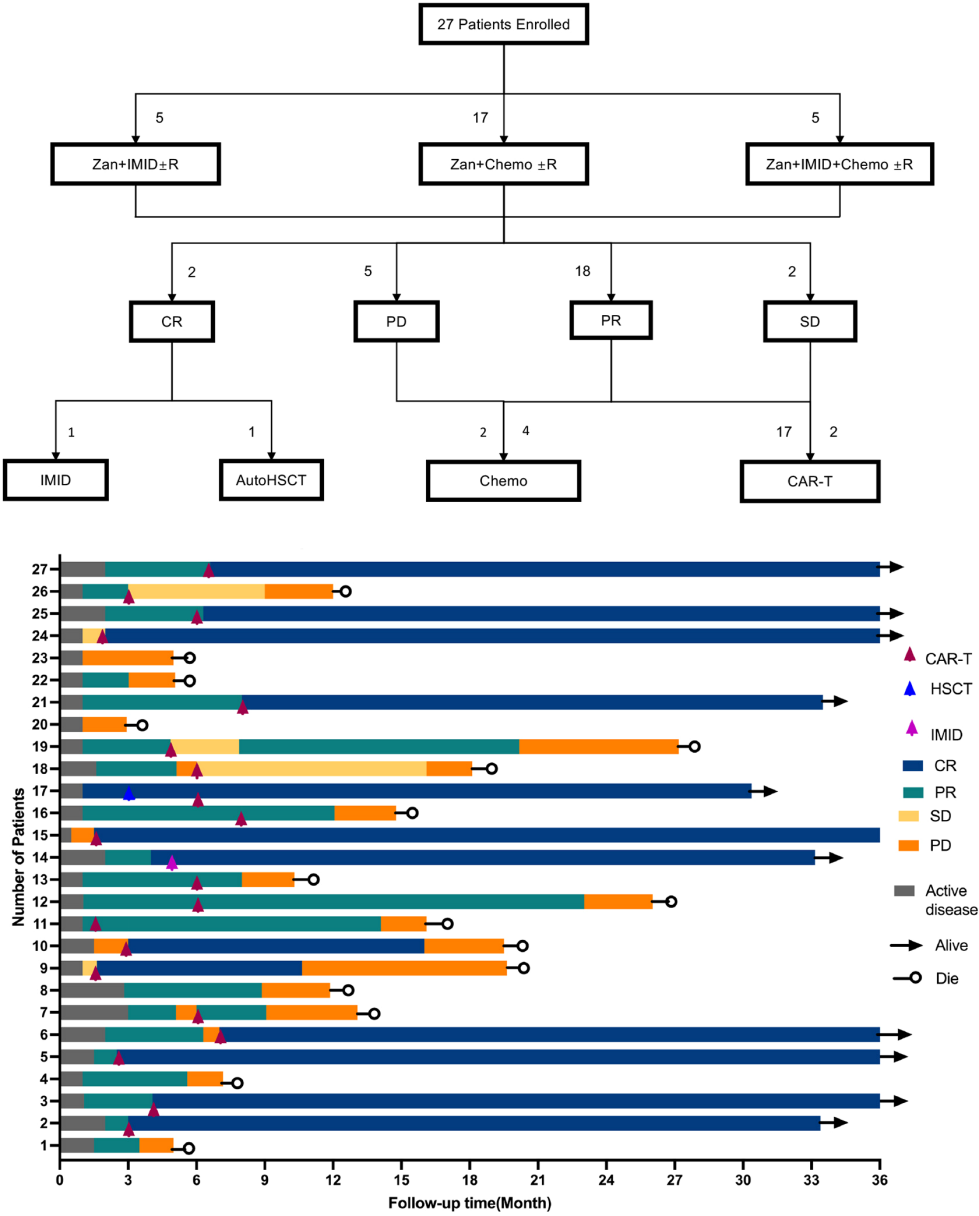
Swimmer plot illustrating individual patient treatment courses and response durations.

**Figure 4:**
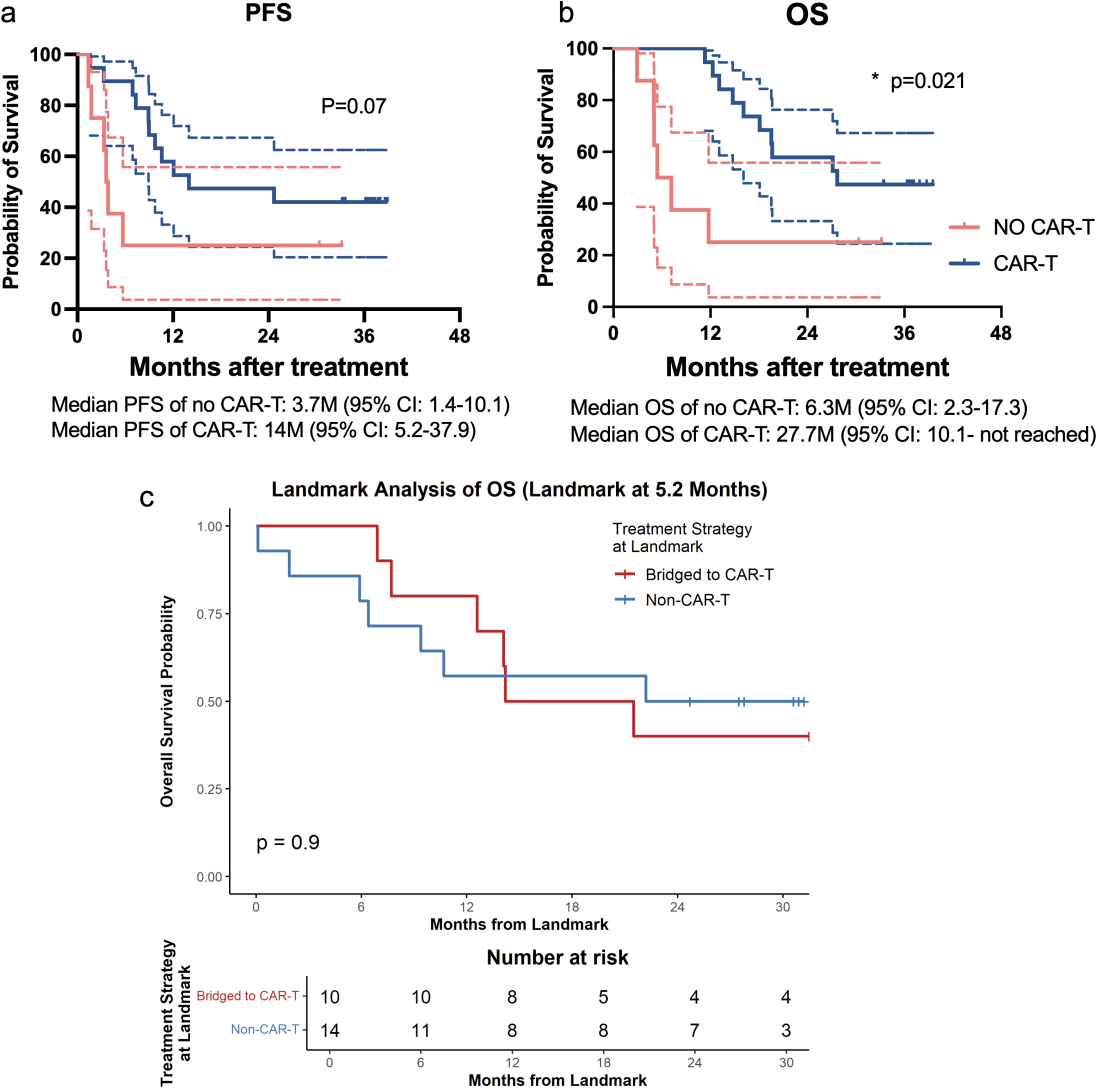
Survival outcomes of CAR-T vs. non-CAR-T cohorts. **(a)** Kaplan-Meier curve for PFS; **(b)** Kaplan-Meier curve for OS. **(c)** Landmark Analysis of OS.

Swimmer plot of individual patient treatment courses and response durations visually illustrates the treatment trajectories, response durations, and survival status of all 27 enrolled patients (**Figure 3**). Patients who achieved CR were either switched to auto-HSCT (1 patient) or maintained on LEN therapy (1 patient). For those with PR, SD, or PD, 19 patients bridged to CD19 chimeric antigen receptor (CAR)-T cell therapy, while the other 6 patients received additional salvage chemotherapy. The ORR and CR in the 19 patients bridged to CAR-T therapy is 89.5% (95%CI: 67.0%-98.2%) and 57.9% (95%CI: 34.5%-78.9%). Median PFS (14 vs. 3.72 months) and OS (27.7 vs. 6.3 months) were associated with longer survival compared to non-CAR-T group (**Figures 4 a, b**). The CAR-T group was associated with improved overall survival relative to the non-CAR-T group (HR = 0.21, 95% CI: 0.05–0.79, P = 0.02).

To mitigate the potential selection bias and immortal time bias inherent in comparing patients who proceeded to CAR-T therapy versus those who did not, we performed a landmark analysis for survival comparison between bridged-to-CAR-T and non-CAR-T groups. The landmark time was set at 5.2 months after initiation of the Zan-based regimen (median Zan-based regimen treatment duration). Patients who experienced a survival event or were censored prior to this landmark were excluded, resulting in 10 patients in the bridged-to-CAR-T group and 14 patients in the non-CAR-T group for this specific analysis. Survival probabilities were calculated from this landmark onward (**Figure 4c**). In the bridged-to-CAR-T group, the survival probability was 80.0% at 12 months post-landmark, followed by a steep decline to 50.0% at 18 months, and stabilizing at 40.0% at 24 and 30 months. In contrast, the non-CAR-T group exhibited an earlier initial drop, with survival decreasing to 57.1% at 12 and 18 months, before stabilizing at 50.0% at 24 and 30 months. Overall, there was no statistically significant difference in survival between the two groups from the landmark timepoint (P = 0.90). These results suggest that while CAR-T bridging may offer better early survival post-landmark, subsequent late events lead to a crossing of the survival curves, ultimately resulting in comparable long-term outcomes in this small subset.

## 4 Discussion

Diffuse large B-cell lymphoma (DLBCL) is a prevalent B-cell lymphoma, with R-CHOP as the standard first-line therapy (21). However, 30%-40% of patients fail to respond due to chemoresistance or relapse, and second-line salvage chemotherapy (e.g., DHAP, GDP, ICE) plus ASCT yields limited outcomes (CR rate <15%) and short median PFS (9, 17, 18, 22). Novel targeted combinations are thus urgently needed for relapsed or refractory (R/R) non-GCB DLBCL. Zanubrutinib, a next-generation BTK inhibitor with higher selectivity and lower off-target activity than ibrutinib, shows superior efficacy in combination with salvage therapy (19, 20, 23).

This study demonstrates that zanubrutinib-combined therapy is effective and well-tolerated in heavily treated non-GCB DLBCL patients. The ORR (74.1%) and median OS (19.6 months) are superior to zanubrutinib monotherapy (ORR: 29.3%; 14) and standard salvage chemotherapy (ORR: 30%; 3), supporting synergistic effects between zanubrutinib and chemotherapy/LEN (24–27). The favorable safety profile is attributed to zanubrutinib high selectivity (13, 28), with hematologic toxicities manageable and consistent with salvage therapy (29–31). The efficacy of the combined therapy seemed to not be affected by most baseline characteristics and was associated with high ORR in high-risk subgroups (IPI ≥3, “Ki-67” ≥80% and ≥3 prior lines treatment). Notably, zanubrutinib-based bridging to CAR-T therapy yielded good outcomes (ORR: 89.5%; median OS: 27.7 months), maybe associated with improved CAR-T efficacy and duration.

Several important limitations of this study should be considered. First, this study was a non-randomized, retrospective analysis. The retrospective design has the inherited selection bias and selection bias is a major concern, as patients were not randomly assigned to treatment regimens. The patients were assigned into different zanubrutinib based regimen by the physician preference, patient frailty, or the previous treatment history. For example, if the patient is fragile, the physician is prone to choose chemo-free regimen. Moreover, the patients who proceeded to CAR-T were typically fitter, had better performance status, and may have been more responsive to the preceding zanubrutinib-based regimen, compared to the patients not proceeded to CAR-T. These baseline differences, rather than the CAR-T intervention itself, could account for the observed survival differences between the groups. Second, the comparison between CAR-T group and non-CAR-T group was subject to immortal time bias. The survival was measured from the initiation of the zanubrutinib regimen, the CAR-T group would inherently have a longer ‘immortal’ period during which no events could occur, artificially inflating their survival estimates. To mitigate this, we performed a landmark analysis with a fixed time point [5.2months, median zanubrutinib treatment duration] and measured survival thereafter. However, landmark analysis cannot fully eliminate bias because some patients were selected for CAR-T after the landmark. Third, this study was small sample size and not randomized. Therefore, given these inherent biases, our findings should be interpreted with caution. No causal inferences can be drawn regarding the effect of CAR-T on survival. Larger prospective randomized controlled trials are needed to validate the findings. Further adjustment for confounders or sensitivity analyses are warranted to validate these observations.

Despite this, zanubrutinib-combined therapy is effective and well-tolerated in heavily treated non-GCB DLBCL patients. It offers a promising treatment option by increasing response rates, overcoming drug resistance, and serving as an effective bridge to CAR-T therapy, with manageable toxicity. Future prospective studies with larger cohorts are needed to validate these findings.

## Data Availability

The raw data supporting the conclusions of this article will be made available by the authors, without undue reservation.
